# Draft Genome Sequence of Highly Rifampin-Resistant *Propionibacterium namnetense* NTS 31307302^T^ Isolated from a Patient with a Bone Infection

**DOI:** 10.1128/genomeA.00819-16

**Published:** 2016-08-11

**Authors:** Guillaume Ghislain Aubin, Stanimir Kambarev, Pascale Bémer, Paul A. Lawson, Stéphane Corvec

**Affiliations:** a Bacteriology and Hygiene Department, Nantes University Hospital, Nantes, France; b EA3826, Laboratory of Clinical and Experimental Therapeutics of Infections, Nantes Medicine School, Nantes, France; c Institut de Recherche en Santé de l’Université de Nantes INSERM U892—CNRS 6299 CRCNA Centre de Recherche en Cancérologie Nantes Angers, Université de Nantes, Team 13: Nuclear Oncology, Nantes, France; d Department of Microbiology and Plant Biology, University of Oklahoma, Norman, Oklahoma, USA; e Institut de Recherche en Santé de l’Université de Nantes INSERM U892—CNRS 6299 CRCNA Centre de Recherche en Cancérologie Nantes Angers, Université de Nantes, Team 2: Clinical and Translational Research in Skin Cancer, Nantes, France

## Abstract

*Propionibacterium namnetense* was recently described as a potential bone pathogen, which is closely related to *Propionibacterium acnes*, a skin commensal microorganism. Here, we report the draft genome sequence of the highly rifampin-resistant strain NTS 31307302^T^ isolated from a patient with a tibia infection.

## GENOME ANNOUNCEMENT

*Propionibacterium acnes* is known as a Gram-positive bacterium constituting a significant part of the human skin microbiota (1). Its natural habitat is mainly the lipid-rich sebaceous glands (2, 3). Acne vulgaris is a common chronic skin disease and is usually associated with the detection of this organism. Moreover, *P. acnes* is more frequently identified in device-related infections (4, 5), producing biofilm in this context (6). Recently, this species has been subdivided into several phylogenetic types that were subsequently afforded subspecies status (7).

We present here the genome sequence of *Propionibacterium namnetense* NTS 31307302^T^ isolated at Nantes University Hospital, France, during a bone infection (8). The isolate showed beta-hemolysis on a blood agar plate and was recently described as being related to *P. acnes* (9). Strain NTS 31307302^T^ is resistant to rifampin, as it has been already reported for *in vitro*-selected mutants (10) or clinical strains of *P. acnes* involved in biofilm or device-related infections (11).

*P. namnetense* NTS 31307302^T^ was grown overnight at 37°C on a Schaedler agar plate (Oxoid, United Kingdom) under an anaerobic atmosphere. Genomic DNA was extracted using a DNeasy blood and tissue kit (Qiagen Gmbh, Germany), according to the provider’s recommendation. A paired-end library was prepared with the NEBNext Ultra DNA library prep kit for Illumina (NEB) and sequenced (2 × 150 bp) on a MiSeq Sequencer (Illumina, USA). *De novo* assembly was performed with Velvet 1.2.10 and VelvetOptimiser 2.2.5 (optimal hash value, 127). A total of 2,846,458 reads were assembled into 24 contigs (15 of them >1 kb) with an average coverage of 127×. Contig reordering and annotation were performed with Mauve 2.3.1 (12) and the NCBI Prokaryotic Genome Automatic Annotation Pipeline (PGAAP) (13), respectively. Average nucleotide identities (ANI) were calculated using Oat 0.91 (14).

The final assembly has a total length of 2,369,664 bp, an *N*_50_ of 626 kb, and a G+C content of 60.5%. About 2,136 coding sequences (CDSs), 46 tRNAs, 84 pseudogenes, three rRNAs, and three noncoding RNAs were revealed by annotation.

To determine genomic differences between *P. namnetense* NTS 31307302^T^ and the closely related *P. acnes* KPA171202, ATCC 11828, and ATCC 6919^T^, we performed a genomic comparison. The draft genome size of the newly sequenced strain is 2.37 Mb, which is 5.02 to 9.81% smaller than *P. acnes* reference strains. The ANI value was 88.5%. Interestingly, our strain showed an ANI value of 99.52% with *P. acnes* SK182B-JCVI (accession no. AFUN00000000.1), recovered during the Human Microbiome Project, which is significantly above the cutoff value of 95% for species delineation (14). Therefore, this strain is likely to be another isolate of this species. Comparing both sequences of the *rpoB* gene, we observed only one point mutation at nucleotide 1319 (G→A) in strain NTS 31307302^T^ leading to an amino acid modification at position 440 (R440H), previously described to be involved in rifampin resistance in *P. acnes* (11).

This draft genome of *P. namnetense* NTS 31307302^T^ will be used for studying virulence features associated with bone infection, especially hemolysin, lipase, or hyaluronidase (15).

### Accession number(s).

The draft sequence of *P. namnetense* NTS 31307302^T^ studied in this project has been deposited at DDBJ/EMBL/GenBank under the accession no. LWHO01000001. The version described in this paper is LWHO01000001.1.
